# Advanced‐stage hepatocellular carcinoma presenting without radiographic liver lesions

**DOI:** 10.1002/ccr3.1480

**Published:** 2018-04-06

**Authors:** Charles Mupamombe, Rajesh Veluvolu, Arslan Ahmad, Mohan Preet, Evelyn Taiwo

**Affiliations:** ^1^ Department of Internal Medicine SUNY Downstate Medical Center 450 Clarkson Ave Brooklyn 11203 New York; ^2^ Department of Gastroenterology SUNY Downstate Medical Center 450 Clarkson Ave Brooklyn 11203 New York; ^3^ Department of Pathology SUNY Downstate Medical Center 450 Clarkson Ave Brooklyn 11203 New York; ^4^ Department of Oncology SUNY Downstate Medical Center 450 Clarkson Ave Brooklyn 11203 New York; ^5^ Department of Oncology Kings County Hospital Center 451 Clarkson Ave Brooklyn 11203 New York

**Keywords:** Gastrointestinal bleeding, gastrointestinal hemorrhage, metastatic hepatocellular carcinoma

## Abstract

In patients with known risk factors for hepatocellular carcinoma and an elevated AFP, the diagnosis should remain on the differential even in the absence of hepatic lesions. High index of suspicion is needed, and aggressive diagnostic approaches are needed to not miss this entity.

## Introduction

Hepatocellular carcinoma (HCC) typically presents with primary hepatic lesions. The most common initial extrahepatic manifestations of HCC reported are bone and lungs. We present a case of recurrent gastrointestinal hemorrhage, with an elevated AFP level, and a perigastric mass with a subsequent final diagnosis of HCC.

Extrahepatic manifestation as an initial presentation of hepatocellular carcinoma (HCC) has been described and well reported. Approximately 15% of cases have extrahepatic manifestations at time of diagnosis, with most common sites being lungs, bone, lymph nodes, and adrenal glands 1. Few cases have been reported to involve the gastrointestinal tract, with hemorrhage due to direct invasion. This presentation confers poor prognosis with an estimated overall survival of 6 months 2. In most cases described, patients present with primary hepatic lesions. Of the few confirmed HCC cases with associated nonvariceal GI hemorrhage, duodenal ulceration has been reported as the most common cause in this presentation 3. The diagnosis of HCC is highly probable with an AFP level >200 ng/mL, and confirmed when imaging shows a hypervascular hepatic lesion >2 cm with an AFP level >400 ng/mL 4. Although rare, HCC cases have been described in patients without cirrhosis or hepatitis 4.

We describe a case of a pathologically confirmed diagnosis of hepatocellular carcinoma in a patient without initial hepatic lesions or cirrhosis, whose main primary presentation was gastrointestinal hemorrhage.

## Case

A 50‐year‐old African American, nonobese man with a history of alcohol abuse for over 26 years, without liver cirrhosis but a history of alcoholic hepatitis, fatty liver disease, chronic pancreatitis, and right‐sided diverticulosis presented with weakness, melena without hematochezia, and stable vital signs. The patient had multiple previous admissions for similar complaints over the preceding 6‐month period and was noted to have AFP level of 210 IU/mL. AFP had been persistently elevated and was being investigated as outpatient. It is unclear why AFP was checked as outpatient due to documentation restrictions, but presumably due to history of alcohol abuse and fatty liver disease. From available records, he had been arranged for follow‐up with a hepatologist, but missed the appointments and hence never obtained a liver biopsy. His PCP noted a downtrend in AFP and, on abdominal MRI, was only significant for hepatomegaly without any masses.

On the first admission, he was found to have a hemoglobin concentration of 9.4 g/dL, hematocrit of 27.3% with an MCV of 96.9 fL, normal renal function, and subsequently discharged from the emergency room with ferrous sulfate for iron deficiency.

Three months later, he returned for similar complaints including lightheadedness and dizziness, vital signs within acceptable limits. His laboratories were remarkable for hemoglobin of 6.9 g/dL, hematocrit 23.6% with MCV 77 fL, and reticulocyte index of 0.5%. Repeat AFP was elevated to 1277 IU/mL, AST 28 U/L, ALT 7 U/L, ALP 94 U/L, and total bilirubin 0.40 U/L. Hepatitis A IgG antibody was positive, IgM negative; hepatitis B surface antigen and antibody were negative; hepatitis C antibody was negative; this suggested a prior hepatitis A infection, but no acute process. His coagulation studies were within normal limits. Fecal occult was positive for blood. The patient required three units packed red blood cells (PRBC) to increase hemoglobin to 9.9 g/dL.

Due to the elevated AFP, testicular ultrasound and *β*hCG level were checked to rule out a nonseminomatous germ cell tumor (NGCT); these were both unremarkable.

**Figure 1:**
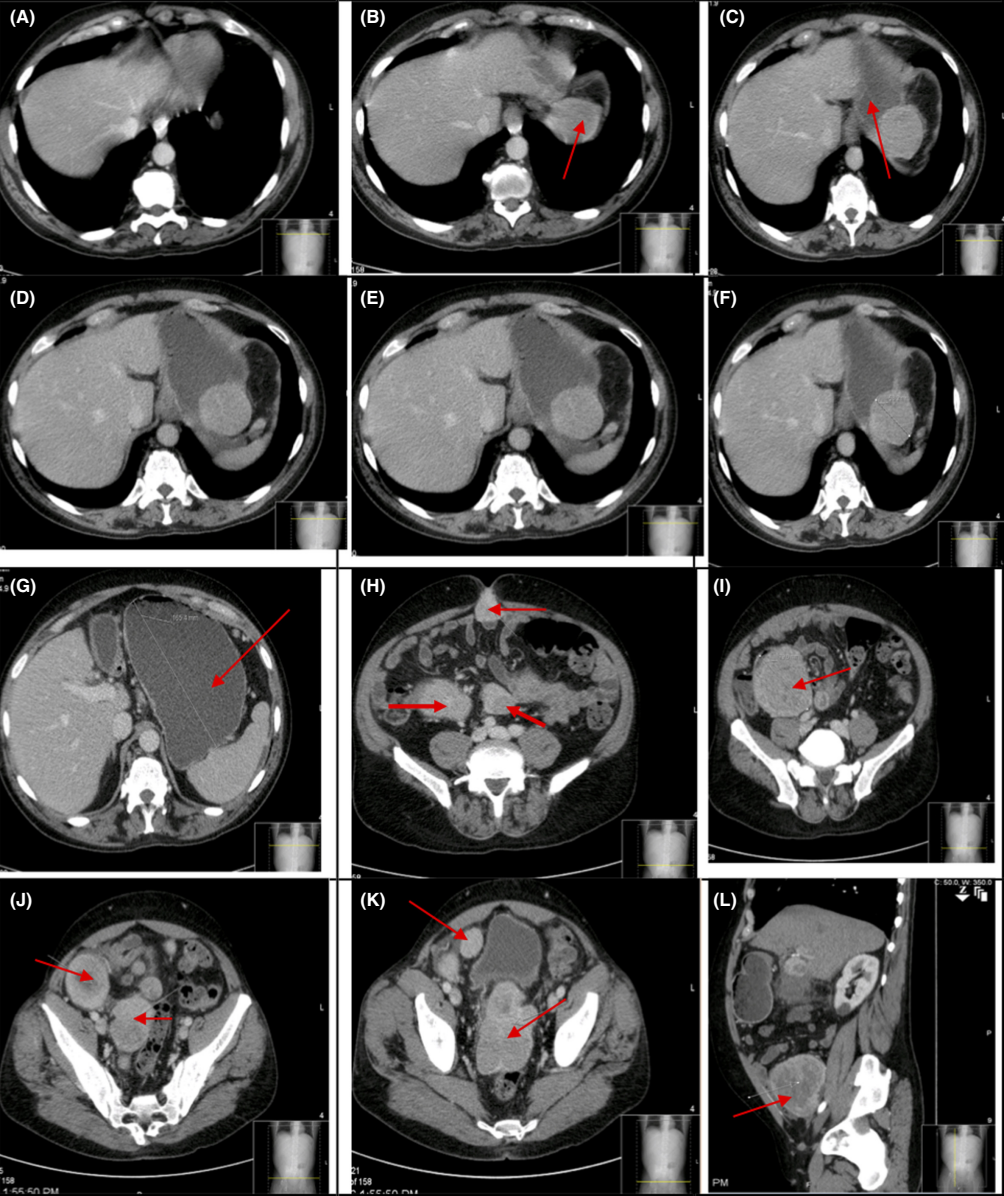
CT abdomen and pelvis after administration of 94 mL of Omnipaque 350 during the arterial phase and portal venous phase. Transverse views are seen A–K. Panels A–G demonstrate different sections of the liver, radiology reviewed as normal in size, and contour with no mass and homogenous attenuation. From Panels B–F, the perigastric mass reviewed as interposed between the left hemidiaphragm and gastric fundus, and measuring 7.6 cm anteroposterior (AP), 5.5 cm transverse, and 5.8 cm craniocaudal. It is marked by an arrow in Panel B. He had extensive fluid in the gastric lumen related to negative oral contrast received prior to examination; this is more prominent in Panel G. Panels H‐K show the multiple abdominal masses (marked by arrows). The largest was reviewed as in the right retroperitoneum, abutting the right psoas muscle, measuring approximately 7.5 cm AP, 9 cm craniocaudal, and 7.5 cm transverse. Panel L show sagittal views of the same CT Scan. Multiple masses can be appreciated, some of which are marked by the arrows.

Hepatic sonography was performed to rule out hepatic pathology causing elevated AFP, which was suggestive of fatty liver disease, with no solid lesions identified. Computed tomography of the abdomen and pelvis in search for further abdominal pathology revealed a 4 cm solid mass, reviewed by radiology as adjacent to or arising from the wall of the stomach which was unchanged from 5 years prior. The reading radiologist suggested the mass may have represented a gastric diverticulum, and the mass appeared smaller on an MRI of the abdomen and pelvis that had been obtained 4 years prior (presumably due to elevated AFP), Figure 1. Review of radiology report dated back 5 years revealed no mention of the perigastric mass; hence, no active investigation had been made. Unfortunately, the images from 5 years prior were not available to us for review. He was discharged and further evaluated by the gastroenterology service as outpatient.

**Figure 2:**
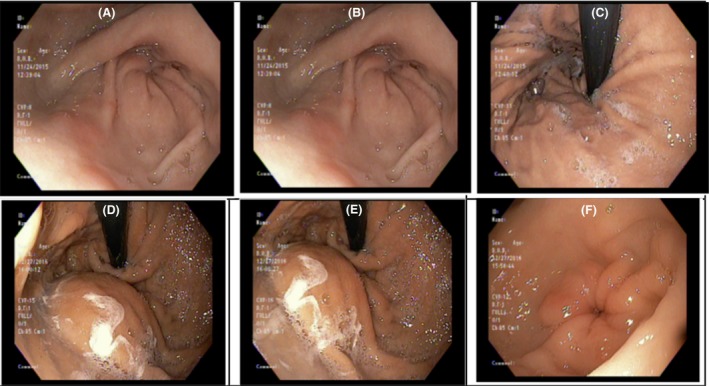
Panels showing selected EGD images. Panels A–C were taken 1 year prior to D–F. Normal mucosa was visualized in both procedures. Panels D–F were suggestive of extrinsic compression. Correlation with CT Imaging, Panels D–F were taken at the time when hepatic masses were visible on CT Imaging. No source of hemorrhage was identified.

**Figure 3:**
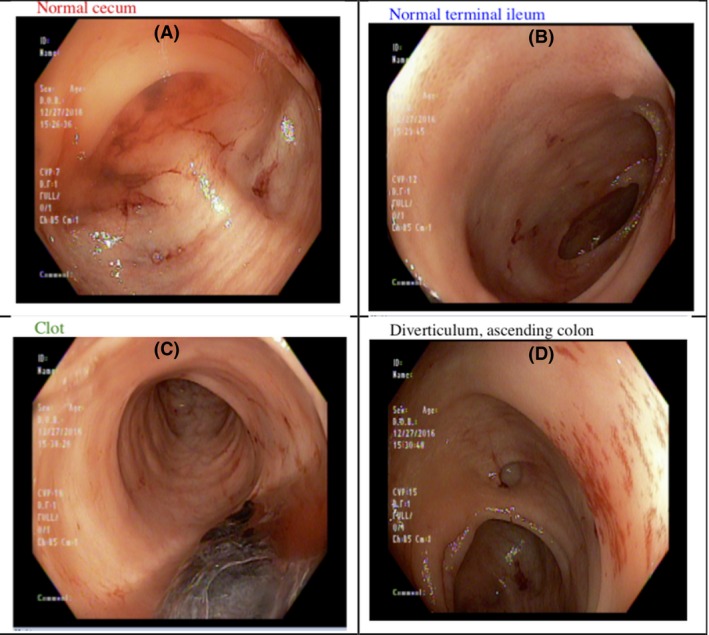
Colonoscopy performed at a time correlating with when hepatic masses first became evident on imaging. Blood can be visualized in panels A–C, but no apparent source or masses. Diverticula are evident on panel D.

An esophagogastroduodenoscopy revealed a 3 cm hiatal hernia, without identifying a source of gastrointestinal bleeding. A capsule endoscopy was performed, but the patient presented a third time as below before results where available. Throughout the patient's care, endoscopy did not reveal a source of hemorrhage. Selected images are shown in Figures 2 and 3.

The patient presented a third time shortly after discharge with worsening weakness and shortness of breath, and black tarry stools. Repeat hemoglobin was 5.5 g/dL. He required additional PRBC transfusion and intravenous proton pump inhibitors for acute gastrointestinal hemorrhage. Repeat CT abdomen and pelvis revealed multiple solid lesions consistent with metastatic disease; again, there were no hepatic lesions. Results of the capsule endoscopy revealed blood loss from the proximal small intestine. An angiogram of the abdominal aorta and its tributaries was unrevealing.

**Figure 4:**
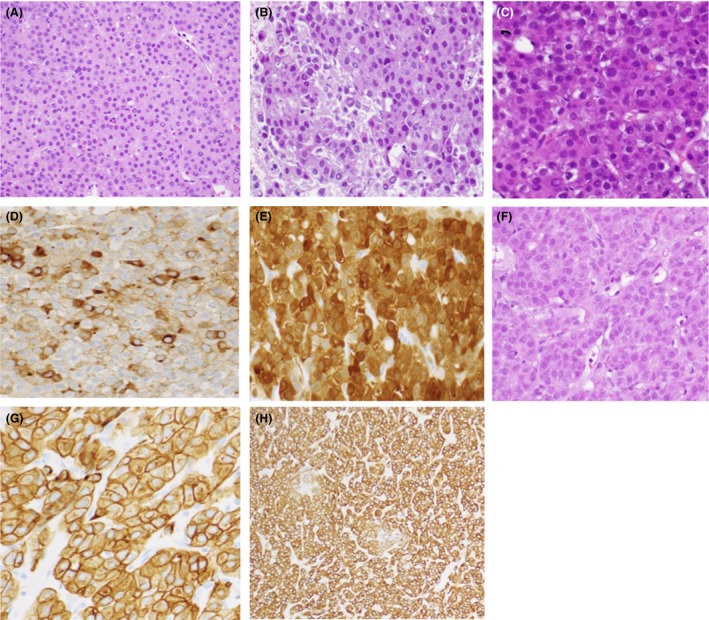
(A) Biopsy of right abdominal mass, correlate with I–J Figure 1. (B) Perigastric aspirate: Image shows pseudoglandular tumor demonstrating pleomorphic tumor cells with brisk mitotic activity. (C) Perigastric lesion biopsy: H&E image shows pleomorphic, hyperchromatic tumor cells with eosinophilic cytoplasm. (D) Perigastric lesion AFP stain: Tumor cells are positive for AFP immunostain. (E) Perigastric lesion arginase stain: Tumor cells are positive for arginase immunostain. (F) Peritoneal implant biopsy: This H&E image shows the same microscopic morphology as the primary abdominal mass lesion. (G, H) Peritoneal implant CAM5.2 and CK8/18 stains: Tumor shows immunoreactivity to low molecular weight cytokeratins.

Due to the presence of the perigastric lesion, perigastric aspirate was performed and initial cytology was suggestive of hepatoid carcinoma (HAC), although subsequent biopsy of the lesion and immunohistochemistry stained negative was for hepatoid carcinoma markers; AE1/AE3, CK19, CK7, CK20, and CDx‐2. The differential diagnosis suggested by pathology was hepatocellular carcinoma. Further extensive immunohistochemistry staining of the perigastric mass revealed positive for hepatocyte, glypican 3, arginase‐1, AFP, CEA (membranous), CD117 (focal), and EMA (focal). The cells were negative for germ cell markers: inhibin, P63, DOG1, SMA, synaptophysin, chromogranin, CK 5/6, CK7, CK20, AE1/AE3, melan A, calretinin, S100, and MOC31. See Figure 4 for biopsies images and stains.

Additional biopsy of abdominal wall lesions was positive for hepatocellular carcinoma (HCC) markers (hepatocyte and glypican‐3 arginase‐1), AFP, *α*l‐antitrypsin, CEA, CAM‐5.2, and CK 8‐18. The sample was negative for germ cell markers SALL‐4, OCT3/4, and PLAP and was also negative for hepatoid carcinoma markers AE1/AE3, CK19, Ck7, CK20, and CDx‐2. Based on the morphology and immunohistochemistry profile, a diagnosis of HCC was determined. An MRI of the abdomen with Liver Protocol was planned but the patient was unable to tolerate the procedure. No liver biopsy was obtained at this point due to lack of radiographic evidence of any lesions.

An attempt to surgically debulk the lesions was unsuccessful, and the patient was subsequently treated for metastatic HCC with sorafenib; a tyrosine kinase inhibitor approved for the treatment of advanced HCC in patients with Child–Pugh A and acceptable in Child–Pugh B patients.

**Figure 5:**
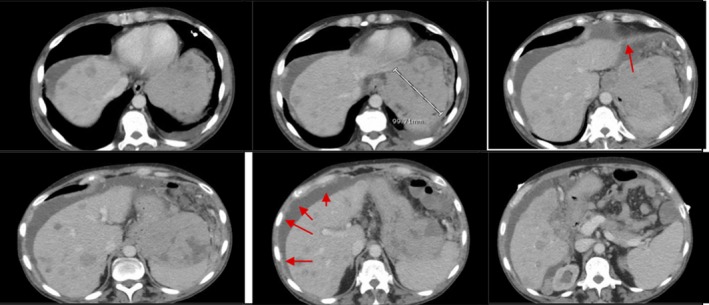
CT scan of the abdomen and pelvis with enteric contrast and intravenous contrast, 96 mL Omnipaque. Only transverse views are shown here. Approximately 7 months after images shown in Figure 1 were taken, a repeat CT abdomen and pelvis revealed new hypodense liver lesions. The above images were taken approximately 11 months after the images in Figure 1 and reviewed as an interval increase in size and number of hypodense hepatic masses. There is also evidence of ascites (arrows). In this image, the perigastric mass also appears to have enlarged.

**Figure 6:**
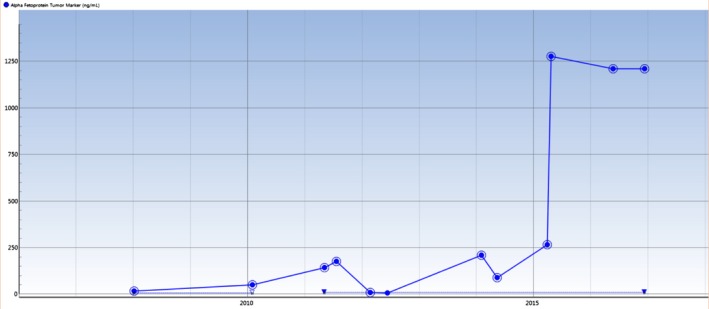
Graph showing the trend of AFP in the patient. A sharp increase is seen post‐2015, approximately at the time of his diagnosis of HCC.

The patient had no significant response to the treatment and developed multiple liver lesions and ascites within 2–3 months of treatment, Figure 5. His functional status deteriorated, and sorafenib was discontinued. At this point, a biopsy of the liver would not have significantly changed the patient's management, and so it was not pursued. A summary of the patient's AFP tumor marker trend is seen in Figure 6. His course was also complicated by the development of cirrhosis and pulmonary embolism, significantly affecting his performance status. The patient expired approximately 2 years from the time of confirmed HCC diagnosis.

## Discussion

Most patients with HCC are diagnosed based on elevated AFP and identifiable liver masses consistent with HCC by multiphase MRI liver findings. The lesions can also be visualized on sonography or CT Scanning. Sonography is particularly used for surveillance and screening in qualifying patients, with various guidelines recommending intervals of 3–6 months in general, although some authors have reported rates of up to 48% of HCC diagnosis with a negative ultrasound study within a year 4. A diagnosis of advanced HCC without liver lesions still remains relatively rare. The differential diagnosis of elevated AFP is broad and includes hepatitis, HCC, cirrhosis, nonseminoma testicular cancer, pure seminoma, gastric cancers, hepatoid carcinoma, pregnancy, and hepatic steatosis 5. Of the differential diagnoses, HAC and HCC closely resemble and can be misdiagnosed for each other.

Generally, HACs metastasize to the liver but can be differentiated from HCC by neighboring cirrhotic lesions and Hep Par‐1‐positive cells which are present in HCC. Metastatic HAC often lacks cirrhotic lesions, and Hep Par‐1 is usually negative 6. Extrahepatic presentation of HCC has been reported in the literature, with the most common site being bone 7. Presentations with omental lesions have also been reported with abdominal pain as the primary presenting complaint 7. Of these, patients usually had an identifiable primary hepatic lesion.

For nonsurgical patients, sorafenib has been shown to prolong median survival and time to progression 8. For advanced HCC with metastases, some studies have shown promise with local treatment modalities including TACE and surgery 9.

Advanced hepatocellular carcinoma is associated with poor prognosis. Overall survival in advanced HCC is approximately 4–6 months 1, 10, of which our patient surpassed, possibly suggesting that extrahepatic HCC behaves differently from the typical intrahepatic HCC. Not much is known on how to manage patients with extrahepatic HCC, and it remains unclear if a difference in tumor biology improves or worsens response to treatment and survival.

Diagnosis of HCC for our patient was delayed, due to lack of hepatic lesions on imaging, and a perigastric aspirate that was not excluding HAC, part of the differential as mentioned earlier. The management of these two entities is different. Treatment‐wise, our patient was a poor surgical candidate due to the deterioration in performance status and was not a good candidate for any local therapy due to multiple masses. He also presented with GI hemorrhage each time, making him an even higher risk patient. He did have a risk factor of alcohol abuse, alcoholic hepatitis, and alcoholic steatosis, which are associated with HCC, even in the absence of cirrhosis 11. This made confirming HCC important due to the implications on management. Interestingly, in all the reported cases of extrahepatic HCC, without liver primaries, the subjects were all hepatitis C positive 11, unlike our patient. Other cases reported of unusual presentations of HCC have had some unusual finding on liver imaging corresponding with the time of or prior to diagnosis, unlike our patient who developed lesions after confirmed diagnosis and treatment had been initiated 12, 13, 14, 15.

In retrospect, it might have been helpful to obtain hepatic protocol images or a liver biopsy to aid in diagnosis, but the lack of hepatic lesions on imaging made such a decision unjustified at the time. Hepatic lesions became apparent only after treatment with sorafenib, at which point biopsy of some of the multiple abdominal implants had been consistent with HCC on pathology, so obtaining a liver biopsy was considered unnecessary. The exact pathology of our patient's recurrent GI hemorrhage was not confirmed. Although capsule endoscopy revealed hemorrhage in the small intestine, an angiogram of the abdominal aorta and its tributaries failed to reveal a source. While GI hemorrhage in hepatic malignancies has been linked to direct tumor invasion in other cases, this was not established in our patient. He also had diverticulosis, a known cause of recurrent lower GI bleed; admittedly, this does not explain the upper GI bleed we found on capsule endoscopy.

## Conclusions from the Case

In patients with risk factors for HCC, this diagnosis should remain part of the differential when the AFP is elevated, even without the usual clinical presentation. In such patients, imaging and tissue biopsies obtained early would play a crucial role in early treatment. Some of these patients, as in our case above, may be challenging to the physician as social factors, such as substance abuse, may lead to poor follow‐up; in this case, patient engagement and education may be crucial. High index of suspicion and aggressive diagnostic strategies are needed in these cases. Close follow‐up should continually be encouraged to these patients.

Our main aim was to add awareness of the difference in presentation of HCC, and more study is needed on this rare presentation to provide better understanding.

## Authorship

CM: Resident Physician for patient, primary author. RV: Gastroenterology Fellow overseeing endoscopy. AA: Pathology Resident Physician reviewing Pathology slides. MP: Primary Oncologist overseeing case. ET: Secondary Oncologist providing second opinion.

## Conflict of Interest

None declared.
